# Supplementary material for: Overexpression of TpGSDMT in Rice Seedlings Promotes High Levels of Glycine Betaine and Enhances Tolerance to Salt and Low Temperature
Source: Biomolecules. 2025 Nov 10;15(11):1576. doi: 10.3390/biom15111576 (PMC12650439; doi:10.3390/biom15111576)
Supplement: Supplementary file 1 [file biomolecules-15-01576-s001.zip › biomolecules-3871657-supplementary.pdf]

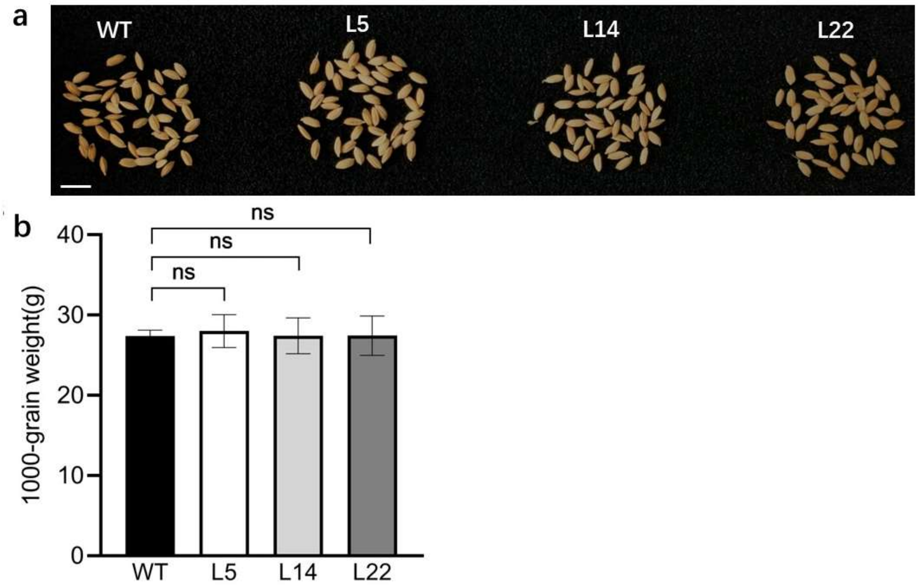

Figure S1. Seed morphology (a) and 1000-grain weight (b) of the WT and T2 transgenic lines(L5,L14 and L22). ns: No significant (Student's *t* test at 0.05), Bar = 1 cm.
