# Supplementary figures and images for: Overexpression of TpGSDMT in Rice Seedlings Promotes High Levels of Glycine Betaine and Enhances Tolerance to Salt and Low Temperature
Source: Biomolecules. 2025 Nov 10;15(11):1576. doi: 10.3390/biom15111576 (PMC12650439; doi:10.3390/biom15111576)

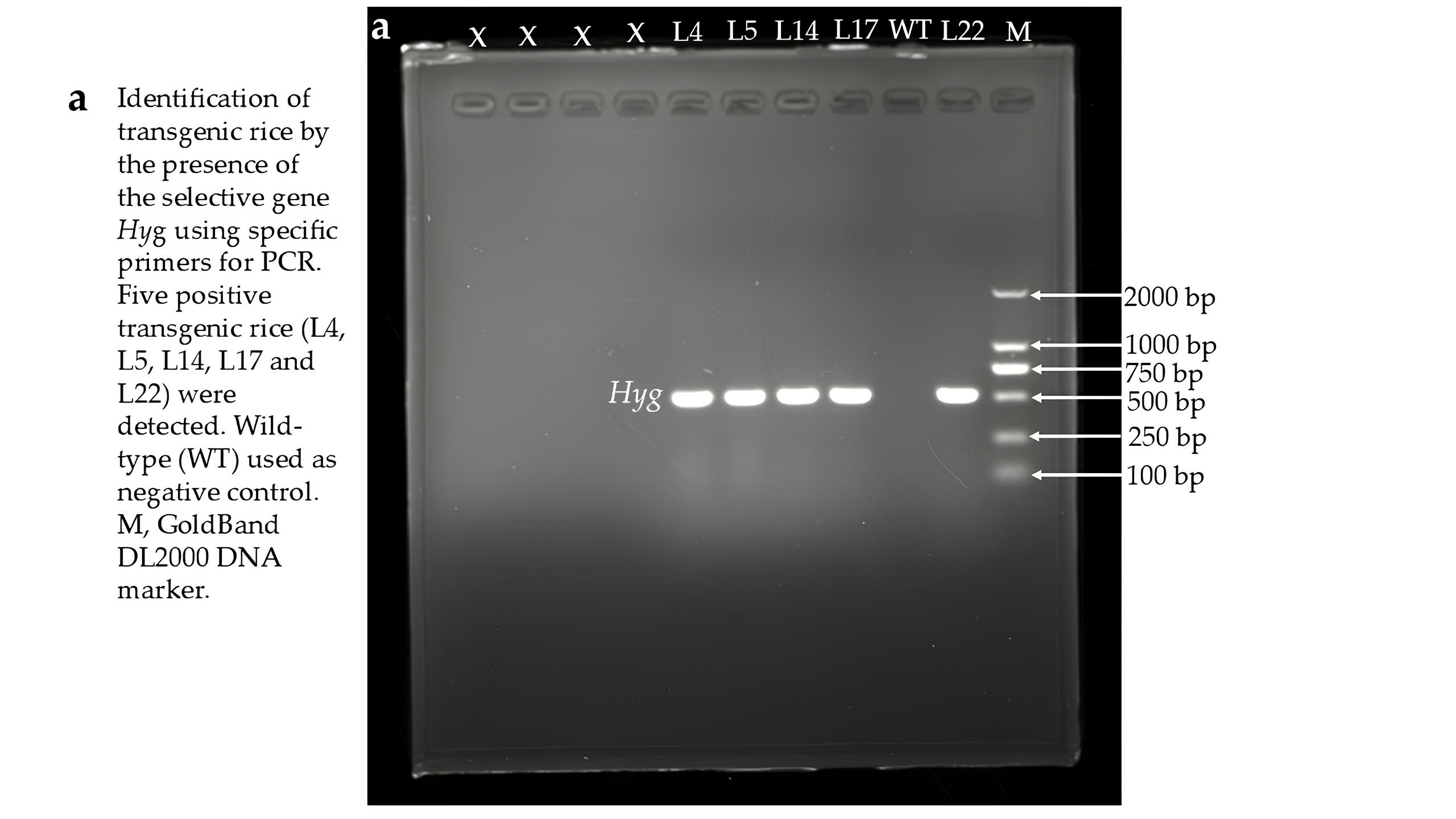

Supplement: Supplementary file 1 [file biomolecules-15-01576-s001.zip › File S1/Original gel image for PCR of gene Hyg.png]

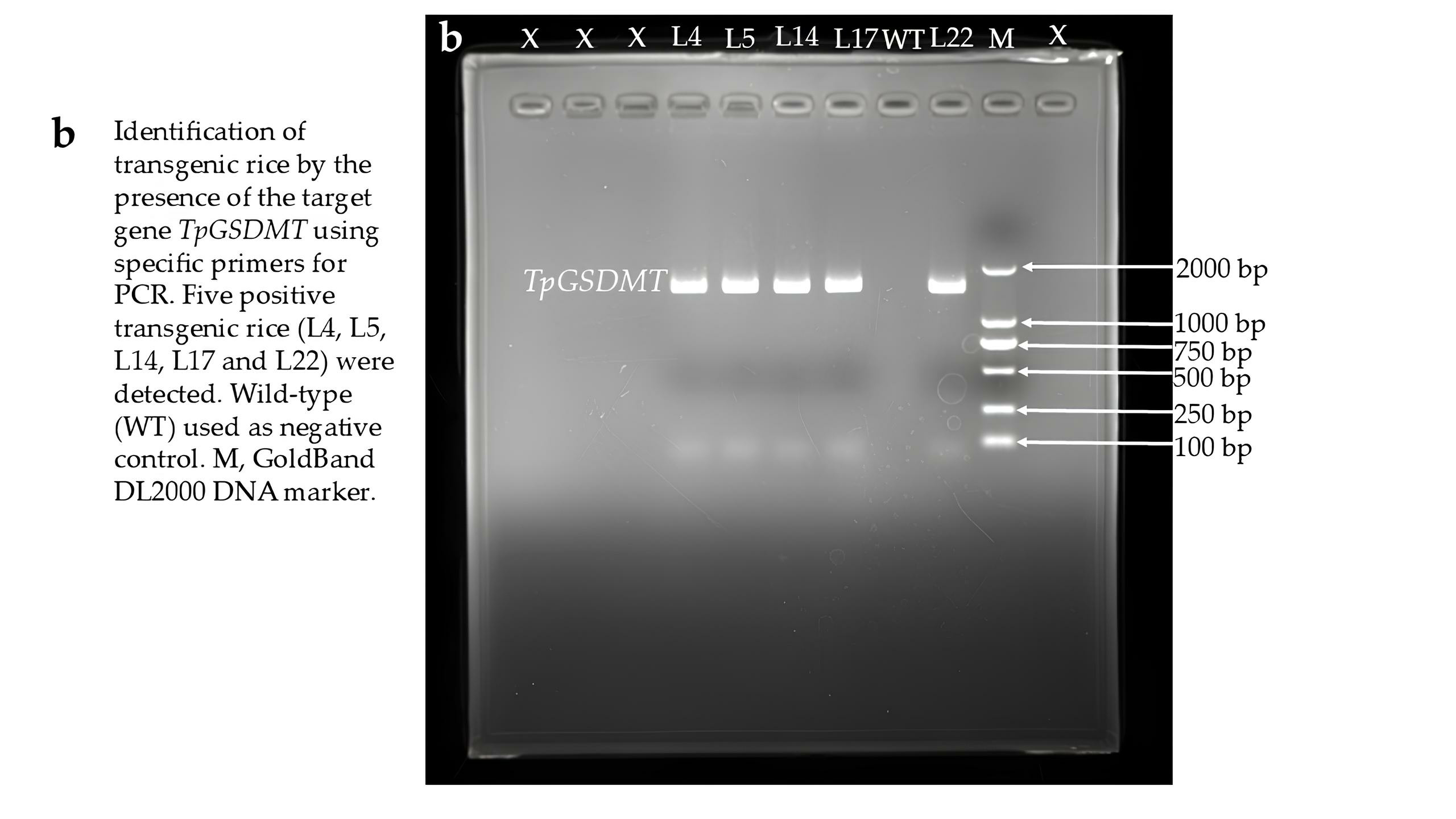

Supplement: Supplementary file 1 [file biomolecules-15-01576-s001.zip › File S1/Original gel image for PCR of gene TpGSDMT.png]
